# Commentary: The changing role of computers in the laboratory

**DOI:** 10.1155/S146392468500013X

**Published:** 1985

**Authors:** Peter B. Stockwell

**Affiliations:** P.S. Analytical Ltd 2 Eagles Drive Tatsfield Westerham Kent TN16 2PB UK


**Journal of Automatic Chemistry**/*Journal of Clinical Laboratory Automation*, **Vol. 7, No. 2 (Apr–Jun 1985), pp. 57–59**

### Commentary: The changing role of computers in the laboratory

**Peter B. Stockwell**
*P.S. Analytical Ltd, 2 Eagles Drive, Tatsfield, Westerham, Kent TN16 2PB, UK*

Over the past few years the use of plasma etching and deposition techniques – so called dry chemistry – has made significant improvements to the production of silicon chips. Smaller scale geometries plus improvements in productivity have ensured the continued reduction in costs for these devices.

Such are the costs involved in providing complete computer systems on a chip, that it is sensible to use them for even modest tasks – for example incorporating them into a telephone keyboard or, more particularly, using them to control a specific instrument function.

It is therefore sensible to include a degree of redundancy in the design of a system such that we could envisage a gas chromatograph as a hierarchy of computers. One computer could convert the gas flows, the oven temperature and parameters, another would linearize a detector etc., with a final computer to co-ordinate the various tasks and act as a means of communicating to the outside world.

Many commercial systems currently available have some similarity to the hypothetical approach. Each of these various computer systems can be transparent to the user. To make the most of today's instrumentation, the analyst does not need to know exactly how each section functions, but he does have to be able to specify the needs of his task and also communicate these to instrument engineers and companies.

In this respect there is still a great deal to be learned since there is a considerable gap in meeting the user's real needs by instrument companies. The analyst will demand certain results and data from his system but this information is of no real interest to the customer for the analytical result. His requirement is for validity of data and answers to specific demands: for example, does the product meet the required specification?

Other influences of chips are still more closely related to analytical chemistry. Recently Ruzicka (*Analytical Chemistry*, **55** [1983], 1041A) described a logical extension to the flow pump manifold designed as a system of integrated conduits situated in a permanent rigid and planar structure.

The grooves forming the flow channels may be imprinted or engraved into a transparent plate and then closed by a flat layer forming a structure of conduits with a hemicircular cross section. The rigidity of the structure ensures repeatability of the dispersion in the sample zone thereby providing further miniaturization of the FIA system.

A further step involves integrating the sample introduction and the detector into one micro conduit thus forming a microchemielectronic device. Fibre optics are used to interface these units to conventional spectrophotometers. The size and cost of these systems will open up vast new fields of applications to the flow-injection approach and transfer the testing from laboratory benches directly on site where it matters most.

Chip technology has also found favour in the clinical laboratory. Eastman Kodak and other companies have described variations on this theme. The salient principles of the former system have been published (H. Curme *et al.*, *Clinical Chemistry*, **24** [1978], 1335) and figure 1 shows a schematic diagram of a chip. This illustrates the chemistry integrated into the multilayers; reference, the determination of urea.

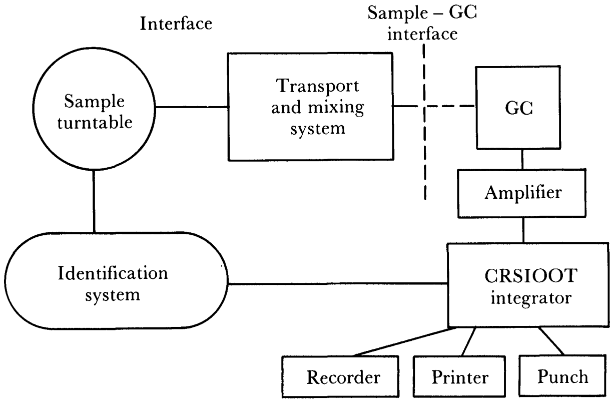

*Figure 1. Schematic diagram of a chip used in clinical laboratory applications.*

The spreading layer is an isotropically porous non-fibrous layer.This metering action compensates for any differences in sample size and serum viscosity. A constant volume per unit area is then applied to subsequent layers. High molecular-weight materials such is protein are removed by this layer and do not interfere. The reactant layer contains the urease which catalyses the hydrolysis of urea in the sample to produce ammonia. Water from the added serum swells the gel, allowing the urease to diffuse into the layer.

A third layer consists of non-ionic materials to pass through to the indicator layer. Ionic compounds are excluded and this provides some degree of selectivity. The indicator layer consists of a gel binder incorporating the indicator reagent. The free ammonia reacts with the indicator to form a dye which has a high molar absorptivity and a broad absorption peak at 520 nm. The reflectance density is measured from the peak at 670 nm.

The final layer is a clear polyester support upon which all the other layers are coated. It is transparent and allows measurement of the colour intensity of the compound formed in the indicator layer.

Performance trials and evaluation tests on the techniques indicate that it is both reliable and accurate. An evaluation was published by Haeckel *et al.* (*JAC*, **1** [1979], 273).

Miniaturization of gas chromatographs and even such devices as mass spectrometers has also been significantly advanced by the space programme.

Analytical chemistry is a great follower of fashion. In the early 1960s, gas chromatography was very much in vogue; in the 1970s the influx of HPLC systems predominated. Towards the end of the 1970s the buzzwords included 'microprocessors' and 'computers'. Now the 1980s threaten to be the domain of the 'robot'.

In such new areas the way is strewn with jargon: for example PCBs, PCs and now LIMS systems. Today's analytical chemists cannot be put off by these symbols. By correctly using the available technology, the chemist can transform his role and provide vital information on the materials under test. To illustrate how analytical chemistry has and continues to change two areas of the subject, let us consider (1) an automated gas chromatograph and (2) laboratory computing systems.

### Automated GC

The changes that have taken place over the years in gas chromatography are predominantly in size and costs but also in terms of response time. Figure 2 shows a schematic diagram of an automatic system developed by the author in the 1970s (*Analytical Chemistry*, **42** [1970], 1136). The emphasis of the development was initially on sample introduction and then, latterly, on data handling and reporting. A detailed description of the interface and transport system has appeared elsewhere (P. B. Stockwell *et al. Proceedings of the Gas Chromatography Meeting of the Institute of Petroleum* [1970], 204); in a sense the approach is still novel because even in the 1980s few analytical systems have resources for sample preparation online and nearly all still use a syringe injection technique.

The data handling aspect has seen marked changes. The system installed in the 1970s had the following components: an Infotronics electronic integrator, a tape recorder, printer and paper tape punch. Chromatographic data could be recorded on magnetic tape from up to four channels and then played back through the integrator to produce a paper tape with retention data and peak areas printed at a faster speed.

This tape could then be analysed further and reports issued from a remote computer system. In this case, the computer was linked by a remote batch terminal to a computer several miles away through a slow speed modem. Analysis packages were written specifically for the task in hand and these programs and the data transferred via paper tape to the computer. Some long time after the results appeared – again in the form of a paper tape before finally the report was printed – very rarely was this report available on the same day as the analyses.

The cost of the data recording system in 1970 was around £11 000, without including rent on the terminal or the cost of computing time. Reports properly computed in a readable fashion were provided from a fully automated system.

In the 1980s gas chromatographs are available which provide many, if not all, of these features as standard. The costs are similar – £11 000 will provide a good system – but the results are directly available, if in some cases the reports are difficult to tailor to particular needs. The chromatography (that is, the separation and resolution) will probably be worse. Also full automation as described above is not available. As this decade advances systems will be made available to cope with special separations and more automation as well as miniaturization.

### Laboratory computing systems

Changes in laboratory computer requirements have been considerably influenced by changes in the chip technol-

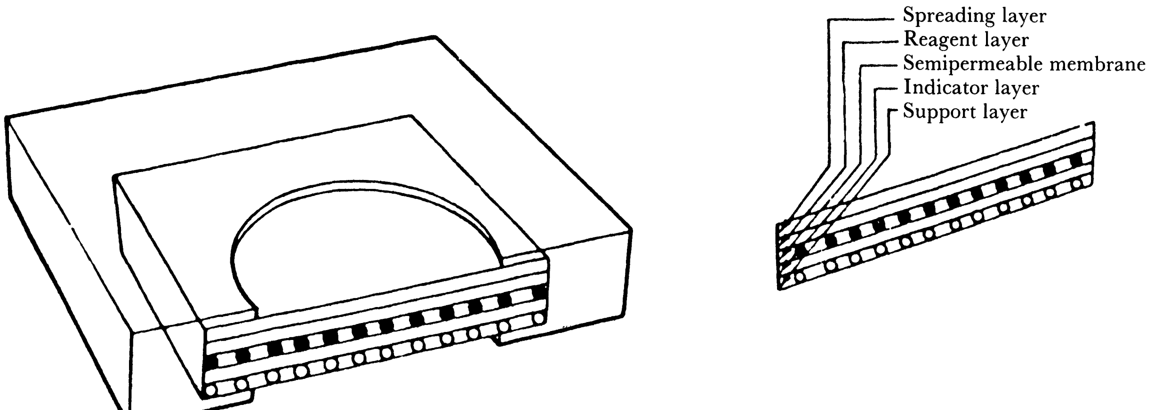

*Figure 2. Automatic gas chromatography: schematic diagrams of a system developed by Dr Stockwell in the 1970s.*

ogy referred to earlier. The changes in the computing requirements at the Laboratory of the Government Chemist in the UK, where the author was Research Director from 1978 to 1980, serve to illustrate the change in emphasis in laboratories.

However, it must be acknowleged that the requirements of any laboratory are extremely influenced by the role that laboratory plays and also by the staff within the organization. A schematic diagram of a computer system to meet laboratory requirements in the 1970s is shown in figure 3.

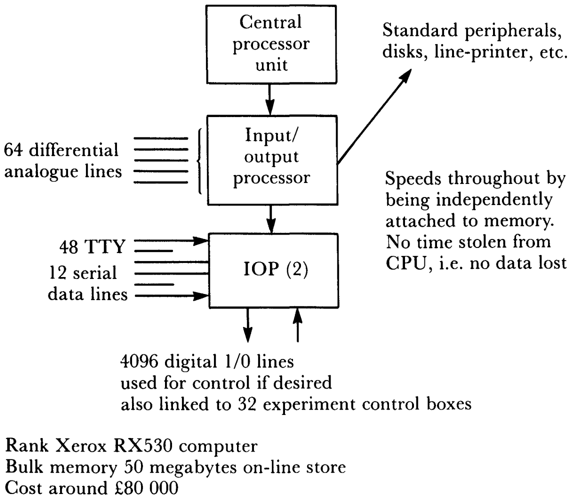

*Figure 3. Schematic diagram of a computer system used for laboratories in the 1970s.*

In essence, the role of the computer was fourfold:

(1) Data-acquisition from laboratory instruments.

(2) Data-reduction and reporting from instruments on-line and off-line.

(3) Batch processing of computer requirements previously carried out on a computer bureau facility.

(4) Data-base management for pattern matching, such as mass special data or for a normal management information system.

A Rank Xerox computer system with 64 Kbyte of core memory and 50 Mbyte of on-line disk store was chosen. The software provided offered a reliable, well-proven operating system. A Fortran compiler, along with the inherent flexibility of the hardware, allowed the appropriate software for the applications envisaged to be developed.

The role of this computer system was modified as the availability of minicomputer and now microcomputer systems was exploited. The system used has been rapidly expanded, and upgraded to provide up to 200 Mbyte of on-line disk store. For each computer linked to the central site, specific software had to be developed for both the mini- and microcomputer and for the central site.

For each application, the software requirements are unique. Very often the coupling to the central site, while extending the capabilities of the instrument itself, was necessitated mainly by the failure of commercial packages to meet the real needs of the users.

In the 1980s with the changing scope and availability of analytical instrumentation, the role of the system changed. An increasing need to reconfigure the system and move away from a centralized system was felt. Today's requirement is for a distributed approach which allows more control at the user side but with the ability to transfer data files as appropriate. A few years ago when the emphasis was placed on microcomputers, the approach I followed was frowned upon; but in 1985 the various marketing claims of the Laboratory Information Management Systems (LIMS) are similar in many ways to a distributed approach.

With today's technology and software we are able to present data in many forms such that our customers can get exactly what they require – communications can be greatly improved. Flexibility in presenting data and speed of response are also vital factors.

The major stumbling block is the difficulty in defining the precise needs of any organization. Some laboratories will have made steps to rationalize their work patterns and have used computer systems. These may well benefit from transferring to a LIMS system. Where no steps have been made along these lines, then I suggest that the managers think hard and long before committing a capital expenditure of £100 000 or more.

Properly specified, a LIMS system will be a considerable bonus to an organization; a system wrongly configured will be disastrous. Before computerizing it is well worth doing a little work simplification. A simplistic approach to computer systems will always pay dividends – for example restricting communications to RS232 lines will reduce the number of headaches involved in linking instruments on line.

Analytical chemistry has certainly changed over the last two decades and it will continue to change rapidly. The analyst has an important role to play in specifying his needs in terms of today's and tomorrow's technology. There are now available devices, chips and instruments, computers and software to tackle many of the tasks in the laboratory. With a good knowledge of what these can achieve – not necessarily exactly how they all work – the analyst can be more able to define his needs to the instrument companies. Robots will find some place in our laboratories but they will never replace the analyst. Also microcomputers will increasingly be integrated into our instruments. But in areas where they have no relevance, they should be avoided.

### Acknowledgement

This article was first published in *Technology Ireland's* January 1985 issue.

